# Supplementary material for: MetaQTL: a package of new computational methods for the meta-analysis of QTL mapping experiments
Source: BMC Bioinformatics. 2007 Feb 8;8:49. doi: 10.1186/1471-2105-8-49 (PMC1808479; doi:10.1186/1471-2105-8-49)
Supplement: Additional File 7 — MetaQTL Package : jar file and tutorial. This Zip archive contains both the MetaQTL JAR file and the files of the tutorial. [file 1471-2105-8-49-S7.zip › org.inra.metaqtl/doc/org/metaqtl/bio/class-use/IBioOntologyTerm.html]

Uses of Interface org.metaqtl.bio.IBioOntologyTerm


|  |  |  |
| --- | --- | --- |
| **Overview** \| **Package** \| **Class** \| **Use** \| **Tree** \| **Deprecated** \| **Index** \| **Help** | |  |
| PREV   NEXT | **FRAMES**    **NO FRAMES**     **All Classes** |  |


---


## **Uses of Interface org.metaqtl.bio.IBioOntologyTerm**

| Packages that use IBioOntologyTerm | |
| --- | --- |
| **org.metaqtl.bio** |  |
| **org.metaqtl.bio.entity** |  |

Uses of IBioOntologyTerm in org.metaqtl.bio

| Methods in org.metaqtl.bio that return IBioOntologyTerm | |
| --- | --- |
| `IBioOntologyTerm[]` | `IBioOntologyTerm.getChildrenTerm()`             Returns the children if the term has chilren, null otherwise. |
| `IBioOntologyTerm` | `IBioOntologyTerm.getParentTerm()`             Returns the parent of the term. |
| `IBioOntologyTerm` | `IBioOntology.getRoot()`             Returns the root of the ontology tree. |
| `IBioOntologyTerm` | `IBioOntology.getTerm(IBioOntologyTerm term)`             Returns if exists the term in the ontology which matches the given one. |
| `IBioOntologyTerm` | `IBioOntology.getTerm(java.lang.String termName)`             This method looks into the ontology if there is a term with the given name and returns it. |

| Methods in org.metaqtl.bio with parameters of type IBioOntologyTerm | |
| --- | --- |
| `IBioOntologyTerm` | `IBioOntology.getTerm(IBioOntologyTerm term)`             Returns if exists the term in the ontology which matches the given one. |
| `void` | `IBioOntology.setRoot(IBioOntologyTerm root)`             Sets the root of the ontology. |

Uses of IBioOntologyTerm in org.metaqtl.bio.entity

| Classes in org.metaqtl.bio.entity that implement IBioOntologyTerm | |
| --- | --- |
| `class` | `OntologyTerm` |

| Methods in org.metaqtl.bio.entity that return IBioOntologyTerm | |
| --- | --- |
| `IBioOntologyTerm[]` | `OntologyTerm.getChildrenTerm()` |
| `IBioOntologyTerm` | `OntologyTerm.getParentTerm()` |
| `IBioOntologyTerm` | `Ontology.getRoot()` |
| `IBioOntologyTerm` | `Ontology.getTerm(IBioOntologyTerm term)` |
| `IBioOntologyTerm` | `Ontology.getTerm(java.lang.String termName)` |

| Methods in org.metaqtl.bio.entity with parameters of type IBioOntologyTerm | |
| --- | --- |
| `IBioOntologyTerm` | `Ontology.getTerm(IBioOntologyTerm term)` |
| `void` | `OntologyTerm.setParentTerm(IBioOntologyTerm parentTerm)`             Sets the parent of the term. |
| `void` | `Ontology.setRoot(IBioOntologyTerm root)` |

---


|  |  |  |
| --- | --- | --- |
| **Overview** \| **Package** \| **Class** \| **Use** \| **Tree** \| **Deprecated** \| **Index** \| **Help** | |  |
| PREV   NEXT | **FRAMES**    **NO FRAMES**     **All Classes** |  |


---
